# Centenarian-Sourced *Lactobacillus casei* Combined with Dietary Fiber Complex Ameliorates Brain and Gut Function in Aged Mice

**DOI:** 10.3390/nu14020324

**Published:** 2022-01-13

**Authors:** Minhong Ren, He Li, Zhen Fu, Quanyang Li

**Affiliations:** College of Light Industry and Food Engineering, Guangxi University, Nanning 530004, China; 1716402006@st.gxu.edu.cn (M.R.); 1816401001@st.gxu.edu.cn (H.L.); fuzhen13@gxu.edu.cn (Z.F.)

**Keywords:** probiotic, antiaging, cognitive function, antioxidant, inflammatory

## Abstract

Dietary intervention could modulate age-related neurological disorders via the gut–brain axis. The potential roles of a probiotic and the dietary fiber complex (DFC) on brain and gut function in aged mice were investigated in this study. *Lactobacillus casei* LTL1361 and DFC were orally administrated for 12 weeks, and the learning and memory ability, as well as the oxidative parameters, inflammatory markers, gut barrier function and microbial metabolite short-chain fatty acids (SCFAs), were investigated. LTL1361 and DFC supplementation ameliorated cognitive ability, attenuated oxidative stress in brain and inflammation in serum and colon, ameliorated gut barrier function, and increased the SCFA concentrations and gene expression of SCFA receptors. The protective effect was more significantly enhanced in aged mice treated with the combination of LTL1361 and DFC than treated with LTL1361 or DFC alone. These results could be associated with the protected morphology of pyramidal nerve cells in hippocampus of mice brain and the downregulation of apoptosis marker caspase-3 in brain and upregulation of tight junction proteins in small intestine and colon. The results indicated that *Lactobacillus casei* LTL1361 and DFC alleviated age-related cognitive impairment, as well as protected brain and gut function. *Lactobacillus casei* LTL1361 and DFC might be used as novel and promising antiaging agents in human.

## 1. Introduction

The proportion of the population aged over 60 years was predicted to exceed 30% in China by 2050, as China is facing the severe challenge of population aging [1]. Aging has been a known major risk factor in many chronic human diseases, such as Alzheimer’s, diabetes, cardiovascular disease, and cancer [2,3,4]. Aging is an inevitable and ubiquitous progress that affects all living organisms, and age-related cognitive decline commonly affects the life of the elderly. Previous studies have reported that the accumulation of free radicals and reactive oxygen species in organism cells cause oxidative stress, resulting in the destruction of tissue and cell structure, dysfunction of the organism, and acceleration of aging [5,6,7,8]. In addition, there is a bidirectional connection between aging and inflammation [9,10]. In the process of aging, the intestinal mucosal barrier function is damaged, and its permeability is increased with the apoptosis of the intestinal epithelium and the thinning of the intestinal mucosa [11]. Harmful microorganisms and toxins generated by metabolism in the intestinal tract reach the circulatory system of the host, causing chronic inflammation and accelerating aging [12].

As aging is inevitable and its relative negative symptoms are complicated, humans pursue antiaging treatments, ranging from diet therapies to drug treatments [13,14,15]. Accumulating evidence has indicated that dietary nutrients (especially dietary fiber) could affect host health by regulating the gut microbiota [16,17,18,19]. Dietary fibers are resistant to digestion and absorption during passage through the stomach and small intestine, but could be fermented in the large intestine by the gut bacteria and could produce beneficial metabolites, such as SCFAs, which are beneficial for host health [20]. SCFA concentrations are likely less than optimal in the elderly, as data indicate that daily dietary fiber intake for the elderly is roughly 40% below the recommended adequate intake [21]. There is also a lower capacity to produce butyrate in the gut microbiota of elderly and lower amounts of bacterial groups which are known as butyrate producers, compared with younger adults [22]. On the other hand, our previous studies revealed that SCFA producers *Roseburia*, Ruminococcaceae, and Clostridiaceae are increased in centenarians living in Bama, Guangxi, with a high-fiber diet (fiber mainly sourced from whole grains and vegetables), and that higher detection frequencies of *Lactobacillus* are significantly correlated with dietary fiber intake [23,24].

Probiotics, as beneficial bacteria, are defined as “live microorganisms that, when administered in adequate amounts, exert health benefits to the host” [25]. *Lactobacillus* is the largest probiotic group that has shown a high possibility of developing functional food. However, individual bacterial strains always exhibit unique bioactivities that require experimental confirmation. Several *Lactobacillus* strains have been reported to have antiaging effects that might be due to their radical-scavenging activity and oxidation stress-attenuating ability [26,27,28,29]. It is reported that probiotic strains derived from the elderly exhibited excellent antioxidant [26], cholesterol-lowering [30,31], and immune-regulating activities [32].

Most previous studies focused on the antiaging effects within a specific disease of treatment with either dietary fiber or probiotics, whereas the evidence is limited with respect to the antiaging effects of centenarian-sourced probiotics combined with the dietary fiber complex. Our previous research indicated that high dietary fiber intake (mainly sourced from coarse cereals, legumes, and dark vegetables) is associated with longevity in Bama [33]. Meanwhile, we successfully isolated *Lactobacillus casei* LTL1361 from the feces of centenarians and demonstrated its potential probiotic properties in vitro in preliminary research. In this study, the antiaging effects of the LTL1361 strain and dietary fiber, as a function of learning and memory ability, antioxidant capacity, inflammation markers, and SCFAs in natural aging mice, were evaluated, which could be useful for the development of synbiotics for the elderly.

## 2. Materials and Methods

### 2.1. Bacteria Strain and Culture

The patented *Lactobacillus casei* LTL1361 strain was isolated from the feces of healthy centenarians living in Bama, China, and preserved at the China Center for Type Culture Collection (CCTCC), with storage number CCTCC M 2,019,018 [34]. The LTL1361 strain was activated on de Man–Rogosa–Sharpe medium (MRS) agar plates. A single colony was inoculated in MRS broth medium and cultured at 37 °C for 14 h in a 50 mL test tube. The strain seed culture (1% inoculation) was scaled up in a 5 L fermenter with MRS broth for 12 h. Then, the LTL1361 cells were harvested by centrifugation at 4000× *g* for 10 min at 4 °C, before washing twice with phosphate-buffered saline. After washing, the LTL1361 cells were resuspended in 0.9% saline and adjusted to a concentration of 1 × 10^9^ cfu/mL for animal experiments.

### 2.2. Preparation of DFC

The ingredients used to extract dietary fiber were purchased from the Bama farmers’ market, including corn, hemp seed, black soybean, *Sonchus oleraceus* (bitter vegetable) *Pachyrhizus* leaf, and pumpkin leaf. The method of extracting dietary fiber from corn, hemp seed, and black soybean is described below. The air-dried sample was ground and sieved to obtain a 60 mesh fraction. Petroleum ether was used to get rid of the fat of the sample, and the ratio of the sample to solvent was 1:20 g/mL. The sample was degreased two times to ensure the maximum removal of lipids. The defatted sample was dispersed in water (1:30 g/mL), and then α-amylase and protease were successively added for enzymatic digestion (2 h) to remove starch and protein. Then, the suspension was heated in a water bath (90 °C) with continuous stirring. After 3h, the suspension was centrifuged (8000× *g*, 15 min). The supernatant and sediment were obtained for further extracting. The supernatant was concentrated with a rotary evaporator (RE-52AA, Shanghai Yarong Biochemistry Instrument Factory, Shanghai, China). The water-soluble dietary fiber was then recovered by precipitation of the concentrated supernatant in four volumes of 95% ethanol and lyophilized using a vacuum freeze-dryer (Shanghai Youpu Industrial Co., Ltd., Shanghai, China). The sediment was washed three times with water and ethanol, and then insoluble dietary fiber was obtained by lyophilization. The method of extracting dietary fiber from *Sonchus oleraceus* (bitter vegetable), *Pachyrhizus* leaf, and pumpkin leaf was the same as above without the fat removal, starch removal, and protein removal steps.

The soluble and insoluble dietary fibers were combined. The dietary fibers extracted from the six ingredients were mixed in specific proportions based on a dietary survey of the elderly in Bama, Guangxi [33]. The mixture was named the “dietary fiber complex” (DFC). The compositions of the DFC were determined, and its total dietary fiber, fat, protein, ash, and water contents were determined according to GB 5009.88–2014, GB 5009.6–2016, GB 5009.5–2016, GB 5009.4–2016, and GB 5009.3–2016, respectively. The DFC contained total dietary fiber (76.1%), water (5.8%), protein (1.9%), lipids (1.5%), and ash (3.9%) (on a dry basis).

### 2.3. Animals and Experimental Design

The protocols and experiments were approved by the Ethics Committee of Guangxi University (Approval No.: GXU-2020-163). Twenty-four male C57BL/6J mice (SPF, 18 months old) were purchased from the Experimental Animal Center of Guangxi Medical University (Production License No.: L20160258SCXK Gui 2020–0003). Mice were housed in conditions of 24 ± 2 °C, relative humidity of 55% ± 5%, and a 12 h light/dark cycle, with free access to food and water. All efforts were made to minimize animal suffering and to reduce the number of animals used. After acclimatization for 1 week, mice were randomly divided into four groups (six mice per group): control group (basal diet, administered saline), DFC group (basal diet with 10% (*w*/*w*) DFC, administered saline), LTL1361 group (basal diet, administered LTL1363), and DFC + LTL1361 group (basal diet with 10% (*w*/*w*) DFC, administered LTL1363). The mice were orally gavaged with a 0.2 mL bacterial suspension or 0.9% saline daily for 12 weeks. The dose of probiotics was similar to other studies conducted in mice [28,29]. A treatment overview is shown in Table 1. The basal diet for mice (in line with Chinese standard “Feed Health Standard” (GB13078-2017)) was purchased from Beijing Keao Xieli Feed Co., LTD. The mice in all groups had free access to food and water. Animal experiments lasted 12 weeks. The general health and wellbeing of the animals was checked daily, and food consumption and body weight were evaluated weekly. At the end week of the experiment, the Morris water maze test was conducted.

### 2.4. Morris Water Maze Test

The Morris water maze test was carried out as described previously [35]. The Morris water maze consists of a large circular pool (120 cm diameter, 45 cm depth). The pool was divided arbitrarily into four equal quadrants (I, II, III, and IV) and filled to a depth of 30 cm with water at 22 ± 2 °C. The water was made opaque with a nontoxic white dye. A submerged platform was centered in one of the target quadrants of the pool and submerged 1 cm below the water surface. The position of the platform was unaltered throughout the training trial sessions. Basic training consisted of a hidden-platform acquisition training session for five consecutive days and a probe trial session. The Morris water maze was conducted on the week before the mice were sacrificed. The tests were assessed by two investigators who were completely blinded to the mouse groups. Each mouse was subjected to four trials per day at an intertrial interval of 120 min. For each trial, the platform was retained at the same place, and the time spent to locate the hidden platform (escape latency) and the path length of each mouse were recorded. On the sixth day, the platform was removed from the water for the probe trial. The number of times that each mice crossed the center of the quadrant (where the platform was previously located) at an interval of 1 min was recorded for the evaluation of memory performance. The swimming orbits and times of test mice were recorded using an overhead camera during the experimental period, and the data were analyzed using Supermaze software (Shanghai XinRuan Information Technology Co., Ltd., Shanghai, China).

### 2.5. Preparation of Tissues and Blood

Mice were fasted overnight before being killed at the end of this experiment (week 12). Blood samples were collected from the orbital vein, and then the mice were sacrificed. Serum samples were collected by centrifugation (2000× *g*, 4 °C, 15 min) and stored at −80 °C until analysis. The brain tissues were excised quickly and carefully. The brain tissues were weighted and divided into two equal parts; the left part of the brain was immediately fixed in 10% formalin for histological analysis, while the right part was snap-frozen in liquid nitrogen and kept at −80 °C until use. Small intestine and colon tissue samples were precisely dissected, flushed, sucked dry, and then weighed as previously described [36]. A ~1 cm section in the middle segment of the small intestine and colon tissues was cut and fixed in 10% formalin for histological analysis. Meanwhile, small intestine and colon tissue and colon contents were collected in sterilized Eppendorf tubes and immediately stored at −80 °C for subsequent analysis. The brain, small intestine, and colon tissue indices were calculated using the following formula: tissue index = tissue weight/body weight × 100%.

### 2.6. Histopathological Analysis

The left part of the brain and the middle segment of small intestine and colon tissues (about 1 cm) were excised and fixed with 10% formalin. The tissues were processed by dehydration, cleaning, infiltration, and embedding for sectioning. Then, the sectioned samples were stained with hematoxylin and eosin (H&E) and observed under a light microscope. Images were captured at 200× magnification for the hippocampal CA1 and CA3 regions, 100× magnification for small intestine tissue, and 40× or 100× magnification for colon tissue.

### 2.7. Measurement of Oxidation-Associated Biomarkers and Inflammatory Cytokines

The whole right part of the brain and the colon tissue sample were weighed and homogenized with cold PBS (PH7.4) at a weight-to-volume ratio of 1 g:9 mL, and then centrifuged (8000× *g*, 4 °C, 10 min) to obtain the supernatant for further analysis. The total antioxidant capacity (T-AOC), malondialdehyde (MDA) content, and superoxide dismutase (SOD) activity of brain and serum were evaluated by chemical colorimetric analysis with the T-AOC assay kit, MDA assay kit, and SOD assay kit (Nanjing Jiancheng Institute of Biotechnology, Nanjing, China), respectively, according to the manufacturer’s protocols. The contents of interleukin-10 (IL-10) and tumor necrosis factor-α (TNF-α) in serum and colon tissue were measured using an enzyme-linked immunoassay with the IL-10 assay kit and TNF-α kit (Shanghai Jianglai Biotechnology Co., Ltd., Shanghai, China), respectively, according to the manufacturer’s instructions.

### 2.8. SCFA Analysis

SCFAs including acetate, propionate, and butyrate were analyzed using an external standard method described by Zhu et al. [37] with minor modifications. Briefly, 0.2 g of colon contents were suspended in 2 mL of saturated NaCl solution. The mixtures were vortexed uniformly for 30 min and then centrifuged at 12,000× *g* for 10 min. The supernatant was acidified with 100 μL of 80% H_3_PO_4_ and extracted with 2 mL of ethyl ether. The contents of SCFAs were determined using an 8890N gas chromatograph with an FID detector (Agilent Technologies, Santa Clara, CA, USA). Separation was achieved using an HP-innowax capillary column (30 m × 0.25 mm × 0.25 µm film thickness, Agilent Technologies Inc.). The injector and detector temperature were both 250 °C. The flow rate of nitrogen carrier gas was kept at 1.5 mL/min. Then, 1 µL of derivatized sample was injected at a split ratio of 10:1. The initial column temperature was 100 °C, ramped to 150 °C at the rate of 8 °C /min, increased to 170 °C at 5 °C /min, and then finally increased to 230 °C at the rate of 30 °C/min, before being kept at this temperature for 2 min.

### 2.9. RNA Isolation and Quantitative Real-Time PCR analysis

Tissue total RNA was isolated using a total RNA Extraction Kit (Solarbio, Beijing, China) following the manufacturer’s instructions, and the concentration was determined using an Infinite M200 pro continuous wavelength multifunctional microporous detector (Tecan, Männedorf, Switzerland). The cDNA was synthesized using a reverse transcriptase kit (Beyotime, Shanghai, China) according to the manufacturer’s instructions. Real-time quantitative PCR was performed using SYBR Green Realtime PCR Master Mix in a Roche LightCycler 96 real-time PCR instrument (Roche Diagnostics Co., Ltd., Basel, Switzerland). Primer sequences of the target genes are listed in Table 2.

Each reaction included 1 μL of template DNA, 7 μL of ddH_2_O, 10.0 μL of 2× ChamQ Universal SYBR qPCR Master Mix (Vazyme, Nanjing, China), and 1 μL of primer 1 and primer 2 with a concentration of 10 μM. Real-time PCR conditions consisted of an initial denaturation step at 95 °C for 60 s and an amplification step, followed by 40 cycles of denaturation at 95 °C for 15 s, annealing at 60 °C for 15 s, elongation at 72 °C for 60 s. At the end of the PCR assay, a dissociation curve analysis was performed to check for nonspecific products. All genes were compared with the housekeeping control gene β-actin using the 2^−∆∆Ct^ calculation method.

### 2.10. Statistical Analysis

All data were expressed as the mean ± standard deviation. Statistical analyses were performed with SPSS V22.0 statistical software for Windows (SPSS Inc., Chicago, IL, USA). Comparisons between groups were performed using an independent-sample *t*-test. The differences among more than two groups were analyzed using one-way analysis of variance (ANOVA) followed by Duncan’s test. Significance was set at *p* < 0.05.

## 3. Results

### 3.1. Effects of Lactobacillus casei LTL1361 and DFC on Learning and Memory of Aged Mice

**Figure 1:**
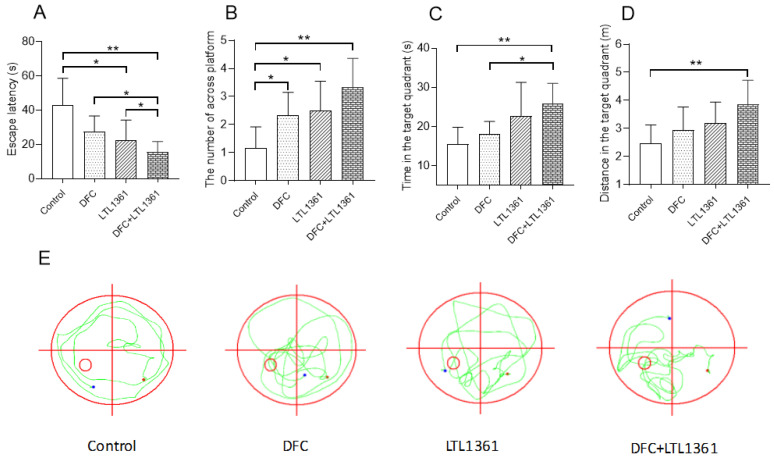
Effects of DFC and *Lactobacillus casei* LTL1361 on swimming performance in the spatial probe test: (**A**) escape latency; (**B**) the number that crossed platform; (**C**) time in the target quadrant; (**D**) distance in the target quadrant. * Significant difference between treatments (*p* < 0.05); ** significant difference between treatments (*p* < 0.01); *n* = 6. (**E**) Representative traces of mice in different groups (the red circle is the exploration area; the green line is the trajectory; the red dot is the starting point; the blue dot is the ending point).

The Morris water maze is widely used to study spatial learning and memory of mice [28,38,39]. The Morris water maze test was carried out after 11 weeks of treatment. The performance of a hidden-platform acquisition training session for five consecutive days in all groups was evaluated, and the results are shown in Table 3. The escape latency of mice in all groups was shortened with the extension of training time. Compared with the first day, the escape latency of the fifth day was significantly decreased in the three treatment groups (DFC group, LTL1361 group, *p* < 0.05; DFC + LTL1361, *p* < 0.01), but not significant in control group (*p* > 0.05). Meanwhile, compared with the control group, the latency of the DFC + LTL1361 group was significantly reduced from the third day to the fifth day, but the latency of the DFC group and LTL1361 group was significantly reduced only on the fifth day. The spatial probe test was conducted on the sixth day, and the results are shown in Figure 1. Compared with the control group, the escape latency of all treatment groups was reduced (Figure 1A), and the number that crossed the platform significantly increased, especially in the DFC + LTL1361 group (Figure 1B). *Lactobacillus casei* LTL1361 combined with DFC significantly prolonged the swim time and distance in the platform quadrant in the spatial probe test (Figure 1C,D). Aged mice of the control group swam centered around the quadrant with an unclear movement direction, but the mice in treatment groups always swam around the target quadrant to search for the platform (Figure 1E). The results indicated that learning and memory ability were impaired in aged mice; however, the DFC and *Lactobacillus casei* LTL1361 could improve the ability of aged mice.

### 3.2. Effects of Lactobacillus casei LTL1361 and DFC on Gene Expression of Apoptosis-Related Protein Markers

**Figure 2:**
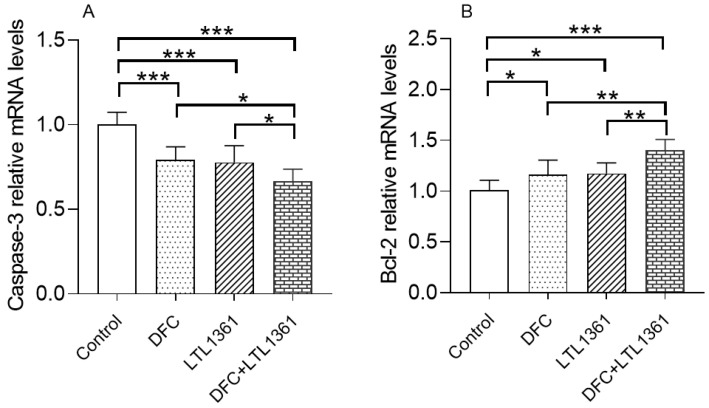
Effects of *Lactobacillus casei* LTL1361 and DFC on the relative mRNA expression of apoptosis markers caspase-3 (**A**) and Bcl-2 (**B**) in the brain tissue (* *p* < 0.05, ** *p* < 0.01, *** *p* < 0.001, *n* = 6).

Apoptosis, a key mechanism of programmed cell death, plays a crucial role in development, degeneration, and regeneration in many body organs, especially the brain [40,41]. The gene expressions of apoptosis-related markers, including proapoptotic protein caspase-3 and antiapoptotic protein B-cell leukemia/lymphoma 2 (Bcl-2), were detected in the brains of aged mice, and the results are shown in Figure 2.

The mRNA expressions of Caspase-3 were significantly decreased in the three treatment groups (*p* < 0.001) (Figure 2A), while expressions of Bcl-2 were significantly increased by the treatment of LTL1361 and DFC (*p* < 0.05), especially by their combination (*p* < 0.001) (Figure 2B).

### 3.3. Effects of Lactobacillus casei LTL1361 and DFC on Hippocampus Histology in Aged Mice

**Figure 3:**
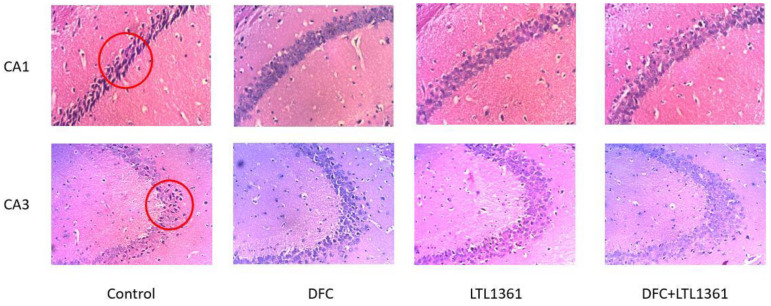
The H&E staining of the hippocampus CA1 region and CA3 region in aged mice brains after intervention in different groups (magnification, 200×). The red circle shows the abnormal morphology of pyramidal nerve cells of the hippocampus.

Evidence has indicated that the hippocampus plays a crucial role in learning and spatial memory abilities, especially the CA1 region [42]. The hippocampal CA1 and CA3 regions of aged mice brains were analyzed by hematoxylin and eosin staining (Figure 3). There was hyperchromic staining with shrinking of pyramidal nerve cells presented in the hippocampus CA1 region of the control group; cells showed abnormal morphology including chromatic agglutination and karyopyknosis. Compared with the control group, the morphology of pyramidal nerve cells improved notably in treatment groups, especially in the DFC + LTL1361 group. The pyramidal cells in the hippocampal CA1 region were arranged more orderly and tightly, the cell morphology and structure were more complete, and chromatic agglutination and karyopyknosis ameliorated obviously. The morphology of pyramidal nerve cells in the CA3 region also improved in treatment groups, but not as notably as in the CA1 region. This indicates that probiotic LTL1361 and DFC could delay the neuron damage caused by aging in the hippocampus of mice brains.

### 3.4. Effects of Lactobacillus casei LTL1361 and DFC on Oxidative Stress

**Figure 4:**
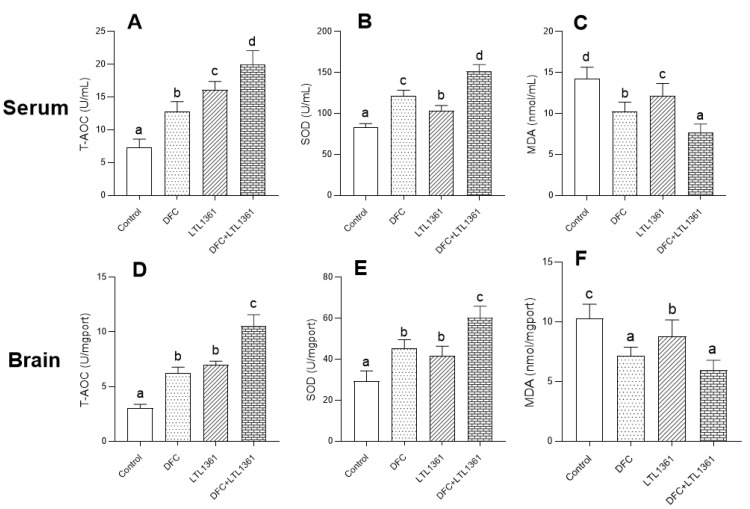
Effects of *Lactobacillus casei* LTL1361 and DFC on T-AOC (**A**,**D**), SOD activity (**B**,**E**), and MDA content (**C**,**F**) in serum and brain. Different superscript letters denote a significant difference in the same index (*p* < 0.05), according to one-way ANOVA (*n* = 6).

Malondialdehyde (MDA) is a byproduct of lipid peroxidation which could describe the level of oxidative stress. To demonstrate the underlying mechanism of the protective effect of *Lactobacillus casei* LTL1361 and DFC in aged mice, the total antioxidant capacity (T-AOC), superoxide dismutase (SOD) activity, and MDA production in the serum and brain of aged mice in different groups were evaluated (Figure 4). Treatment with LTL1361 and DFC significantly enhanced T-AOC level and SOD activity in serum and brain compared with the control group (*p* < 0.05) (Figure 4A,B,D,E). On the other hand, the contents of MDA in serum and brain were significantly decreased in the treatment group (Figure 4C,F). Thus, treatment with *Lactobacillus casei* LTL1361 or DFC could ameliorate oxidative stress in aged mice, and the effect was better in the *Lactobacillus casei* LTL1361 + DFC group than the groups supplemented with *Lactobacillus casei* LTL1361 or DFC.

### 3.5. Effects of Lactobacillus casei LTL1361 and DFC on Inflammatory Markers

**Figure 5:**
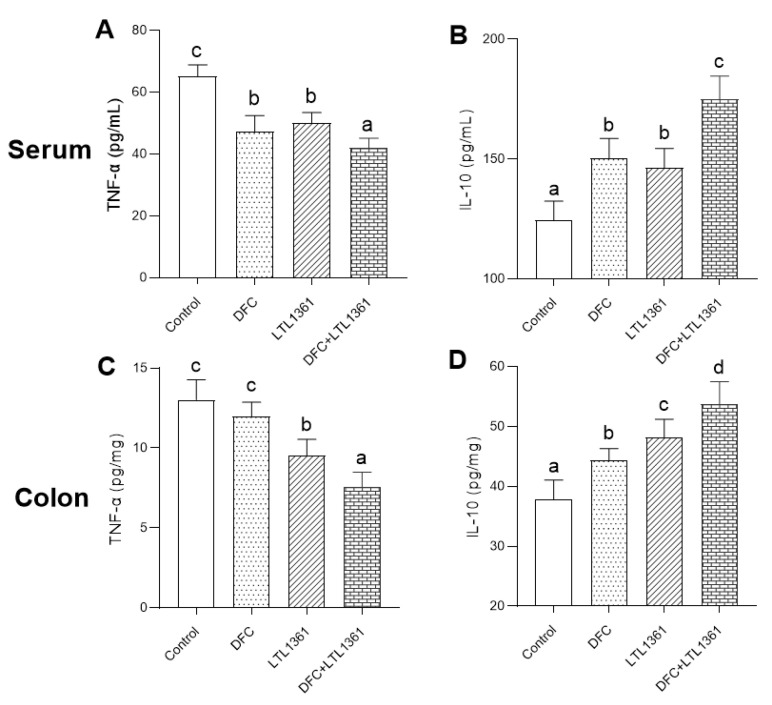
Inflammatory cytokine level in serum and colon tissue in different group after 12 weeks of treatment: TNF-α in serum (**A**) and colon (**C**); IL-10 in serum (**B**) and colon (**D**). Different superscript letters denote a significant difference in the same index (*p* < 0.05), according to one-way ANOVA.

To evaluate the effect of LTL1361 and dietary fiber on the level of inflammation in aged mice, concentrations of proinflammatory cytokine tumor necrosis factor-α (TNF-α) and anti-inflammatory cytokine interleukin-10 (IL-10) in serum and colon tissue were examined (Figure 5). After 12 weeks of probiotic and dietary fiber intervention, TNF-α concentration in serum and colon tissue was significantly decreased in the three treatment groups compared with the control group (*p* < 0.05), whereas IL-10 levels significantly increased (*p* < 0.05). The results indicated that both *Lactobacillus casei* LTL1361 and DFC treatments could increase the level of anti-inflammatory factors and decrease the level of proinflammatory factors in aged mice. Furthermore, it should be noted that *Lactobacillus casei* LTL1361 exhibited a better ameliorative effect with respect to inflammation in colon tissue than DFC, and this was enhanced by combination with DFC.

### 3.6. Effects of Lactobacillus casei LTL1361 and DFC on Gut Barrier Function in Aged Mice

**Figure 6:**
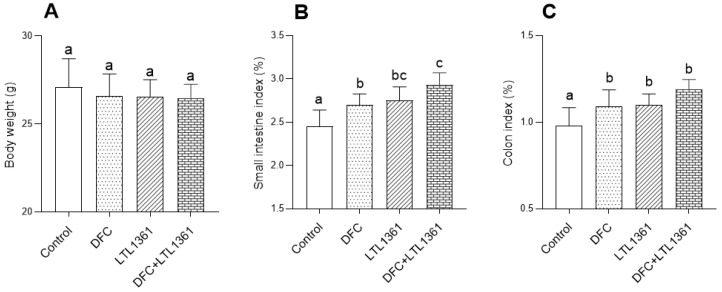
Effects of *Lactobacillus casei* LTL1361 and DFC on body weight (**A**); small intestine index (**B**) and colon index (**C**) of aged mice after 12 weeks of treatment. Gut index was calculated according to the following formula: (tissue weight/body weight), × 100%. Different superscript letters denote a significant difference in the same index (*p* < 0.05), according to one-way ANOVA.

**Figure 7:**
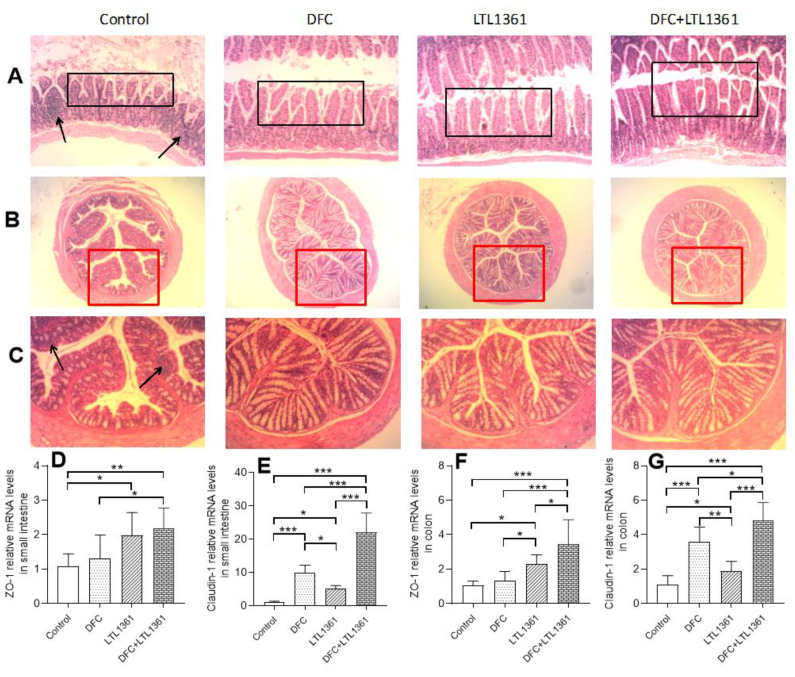
Effects of *Lactobacillus casei* LTL1361 and DFC on gut barrier function in aged mice. (**A**) Representative H&E-stained small intestine section at 100× magnification; representative H&E-stained colon section at 40× magnification (**B**) and 100× magnification (**C**); relative mRNA expression of ZO-1 in small intestine (**D**) and colon (**F**); relative mRNA expression of Claudin-1 in small intestine (**E**) and colon (**G**). Arrows show inflammatory cell infiltration; the black squares show the length of the small intestinal villus; the red squares show the area exhibited in 100× magnification images; * *p* < 0.05, ** *p* < 0.01, *** *p* < 0.001.

Considering the decreased inflammatory level in colon tissue of aged mice in treatment groups, the gut barrier function was assessed. In this study, there were no significant differences in the food consumption between groups. The body weights of mice were slightly reduced in treatment groups with 12 weeks of intervention compared with the control group, albeit not significant (Figure 6A). On the other hand, the small intestine index (Figure 6B) and colon index (Figure 6C) were significantly increased in the three treatment groups. Small intestine and colon samples were stained with H&E to examine morphological changes (Figure 7). Representative pictures indicated that the length of the small intestinal villus obviously increased while inflammatory infiltration decreased in all treatment groups. Similarly, decreased inflammatory infiltration was also observed in colon in the three treatment groups. Tight junction proteins play an important role in maintaining epithelial barrier function [43,44]. The mRNA expressions of genes encoding tight junction proteins, including Claudin-1 and zonula occludens-1 (ZO-1), were investigated. Compared with the control group, the mRNA expression of ZO-1 was significantly upregulated in small intestine and colon tissues in the LTL1361 and DFC + LTL1361 groups (Figure 7D,F). Meanwhile, the mRNA expression of Claudin-1 in the small intestine was upregulated ~10-fold in the DFC group, ~5-fold in the LTL1361 group, and ~22-fold in the DFC + LTL1361 group (Figure 7E), whereas, in the colon, it was upregulated ~3.6-fold in the DFC group, ~1.9-fold in the LTL1361 group, and ~4.8-fold in the DFC + LTL1361 group (Figure 7G).

### 3.7. Effects of Lactobacillus casei LTL1361 and DFC on SCFA Concentration and mRNA Expression of SCFA Receptors in Aged Mice

**Figure 8:**
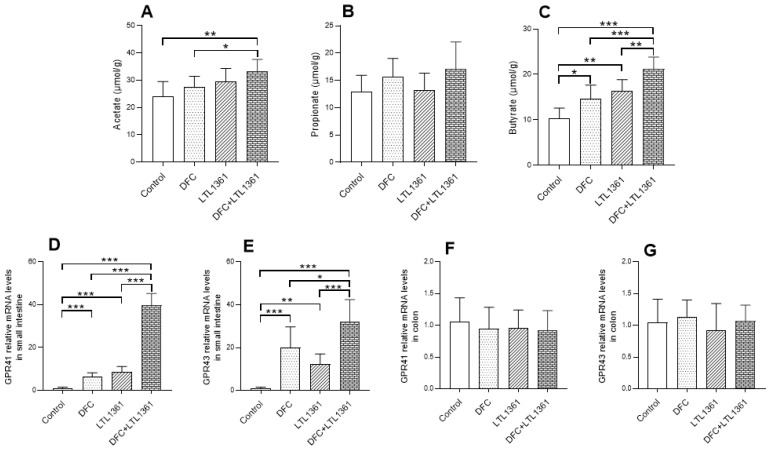
Effects of *Lactobacillus casei* LTL1361 and DFC on SCFA concentrations in colon contents and relative mRNA expression of SCFA receptors in aged mice. Acetate (**A**), propionate (**B**), and butyrate (**C**) concentrations in colon contents. Relative mRNA expression of GPR41 in small intestine (**D**) and colon (**F**); relative mRNA expression of GRP43 in small intestine (**E**) and colon (**G**); * *p* < 0.05, ** *p* < 0.01, *** *p* < 0.001.

SCFAs were recognized as the key microbial metabolites in the gut, which could exert multiple beneficial effects on the host [45]. Given that the level of inflammation was markedly decreased by LTL1361 and DFC treatments in colon tissue, the SCFA concentration of colon contents in different groups was detected (Figure 8). The results showed that concentrations of acetate and butyrate were increased in mice fed with LTL1361 and DFC relative to those fed with a control diet, particularly butyrate (Figure 8C, *p* < 0.05). The contents of propionate did not exhibit a significant difference across groups (Figure 8B, *p* > 0.05).

SCFAs act as endogenous ligands for G-protein-coupled receptors (GPCRs) to exert effects in the organ, and the best-studied SCFA receptors are GPR41 and GPR43, which were later renamed free fatty-acid receptor 3 (FFAR3) and FFAR2, respectively [46]. The mRNA expressions of GPR41 and GPR43 in small intestine and colon tissues were detected. Compared with the control group, the mRNA expression of GPR41 in the small intestine was upregulated ~6-fold in the DFC group, ~9-fold in the LTL1361 group, and ~40-fold in the DFC + LTL1361 group, whereas the mRNA expression of GPR43 was upregulated ~20-fold in the DFC group, ~12-fold in the LTL1361 group, and ~32-fold in the DFC + LTL1361 group. On the other hand, there was no significant difference in the mRNA expression of GPR41 and GPR43 in colon between the control group and treatment groups. These findings suggest that *Lactobacillus casei* LTL1361 and the DFC might have a synergistic effect on upregulating the expression of SCFA receptors in the small intestine.

### 3.8. Correlation among SCFAs, Oxidative Stress, and Inflammatory Markers

**Figure 9:**
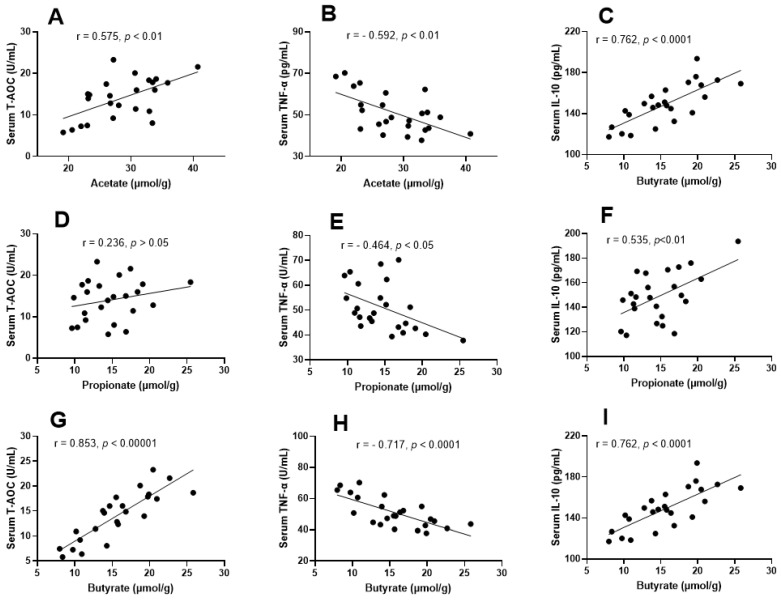
Correlations among SCFAs, antioxidant capacity, and inflammatory markers level. Pearson’s correlations between acetate and serum T-AOC (**A**), TNF-α (**B**), and IL-10 (**C**); correlations between propionate and serum T-AOC (**D**), TNF-α (**E**), and IL-10 (**F**); correlations between butyrate and serum T-AOC (**G**), TNF-α (**H**), and IL-10 (**I**); (inset) Pearson’s correlation coefficient (*r*) and the corresponding *p*-value.

SCFA concentrations, serum oxidative stress, and inflammatory markers were significantly improved with *Lactobacillus casei* LTL1361 and dietary fiber treatment. Therefore, a correlation analysis was performed among SCFA concentration, serum antioxidant capacity, and inflammatory markers. As shown in Figure 9, SCFA concentrations were significantly correlated with serum T-AOC, TNF-α, and IL-10. Acetate, propionate, and butyrate were all negatively correlated with proinflammatory marker TNF-α (*p* < 0.05) (Figure 9B,E,H), but positively correlated with anti-inflammatory marker IL-10 (*p* < 0.01) (Figure 9C,F,I). Acetate and butyrate were highly positively correlated with T-AOC (*r* = 0.572, *p* < 0.01; *r* = 0.843, *p* < 0.00001) (Figure 9A,G).

## 4. Discussion

Aging is associated with a high prevalence of chronic diseases, disability, and cognitive decline [47]. Cognitive abilities, such as memory and learning ability, play a crucial role in the daily functioning of the elderly. Dietary fiber is well recognized for supporting gastrointestinal, immune, and metabolic health [48,49,50], but the appreciation for its importance in cognitive function is less explored with only a handful of observational and interventional studies available [51,52,53]. Recent studies have found that probiotic supplementation, which contained *Lactobacillus casei*, *Lactobacillus plantarumim*, and *Lactobacillus paracasei*, improved learning and memory abilities in d-galactose-treated aging mice [28,29,39]. In this study, the Morris water maze test was conducted to evaluate the effects of DFC and *Lactobacillus casei* LTL1361 on cognitive abilities in aged mice. Escape latency was significantly reduced, and the cumulative duration of the target quadrant was significantly increased in naturally aged mice treated with DFC or *Lactobacillus casei* LTL136. The results revealed that the administration of both DFC and centenarian-sourced *Lactobacillus casei* LTL1361 significantly improved learning and memory ability, and the improving effects enhanced with their combination (Figure 1).

Apoptosis, or programmed cell death, is a fundamental feature of all animal cells which is involved in cell growth, division, and differentiation, and which causes a series of changes in cell morphology and function [40]. In recent years, caspases were found to be highly associated with cell apoptosis. Among these, caspase-3 is a common downstream effector of apoptotic pathways and a core protease that mediates cell apoptosis [54,55]. The mRNA expression of caspase-3 in the brain was significantly downregulated in mice administrated with DFC, *Lactobacillus casei* LTL1361, and their combination for 12 weeks, but the mRNA expression of antiapoptotic protein Bcl-2 was significantly upregulated (Figure 2). The results indicated that DFC and *Lactobacillus casei* LTL1361 could ameliorate apoptosis in the brain of aged mice. Furthermore, the hippocampus of aged mice brains was analyzed by H&E staining. Neuropsychological studies with hippocampal amnesia have provided evidence that the memory system is critically dependent on the hippocampus, especially the CA1 region [42,56]. The morphology of pyramidal nerve cells in the CA1 region improved notably in treatment groups compared with the control group. We speculated that the ameliorated effects of cognitive abilities might be associated with the protective effect of pyramidal nerve cell morphology in the hippocampal CA1 region (Figure 3).

Evidence has indicated that cumulative oxidative stress plays a role in aging [9,57,58]. Dietary fiber and probiotic supplementation have been reported to reduce oxidative stress [28,29,59,60,61,62]. The imbalance between antioxidants and pro-oxidants is called oxidative stress, and it is related to various diseases [63]. Previous studies have reported that mitochondrial reactive oxygen species and free radicals play critical roles in cellular oxidative damage [58]. In vivo, free radicals act on lipids to produce a peroxidation reaction, and the final oxidation product is MDA, which indicates increased oxidative stress [7,64]. The total antioxidant capacity (T-AOC) consisting of enzymes such as SOD, glutathione peroxidase (GSH-Px), and catalase (CAT), as well as nonenzymatic compounds (e.g., glutathione, vitamin E), can prevent the oxidative damage of lipids [64]. A previous study found that a probiotic mixture could significantly increase the activities of SOD and CAT, as well as decrease MDA content, which eventually ameliorated the cognitive deficit in a d-gal-induced aging mouse model [28]. Similarly, our results also revealed that administration of dietary fiber or LTL1361 and their combination significantly enhanced T-AOC level and SOD activity and decreased MDA concentration in both serum and brain tissue compared with the control group; these protective effects might be associated with the amelioration of learning and memory function.

Increased levels of inflammatory markers are related to aging-associated pathologies [65]. TNF-α is a proinflammatory cytokine involved in systemic inflammation, which is mainly secreted by macrophages and monocytes [66]. Increased levels of TNF-α usually aggravate the degree of inflammation in the body. On the other hand, IL-10 is an anti-inflammatory factor that plays roles in downregulating the inflammatory response and antagonizing inflammatory mediators [67]. Evidence has shown that dietary and probiotic interventions affect host health and aging by improving immune homeostasis and suppressing chronic inflammation [68,69]. In this study, TNF-α concentration was significantly decreased and IL-10 was significantly increased in both serum and colon tissue of aged mice following 12 weeks of probiotic and DFC intervention, compared with the control group. Meanwhile, the small intestine index and colon index were significantly increased in the three treatment groups. Colon histology revealed significant inflammatory infiltration within the colon lamina propria of aged mice in the control group, while aged mice in the three treatment groups exhibited relatively lower inflammatory infiltration. Such an effect was also observed in the small intestine. The results demonstrated that intestinal inflammation occurred during the aging process, and that DFC or *Lactobacillus casei* LTL1361 had the potential to limit gut inflammation. Previous studies have indicated that intestinal inflammation is related to epithelial barrier function [70,71]. The gut barrier integrity and function are compromised during the aging process, which includes increased epithelial tight junction permeability and decreased mucus production; ZO-1 and Claudin-1 are important indicators of intestinal barrier function [11,43]. In this study, the mRNA expression of ZO-1 and Claudin-1 was significantly upregulated in both small intestine and colon tissues in treatment groups, especially in the group with the combination of DFC and *Lactobacillus casei* LTL1361, indicating a potential beneficial change in gut permeability. Therefore, we speculated that DFC and *Lactobacillus casei* LTL1361 improved the maintenance of mucosal barrier integrity and immune homeostasis.

Previous studies have shown that SCFAs can promote intestinal epithelial cell proliferation [72,73]. Butyrate is an essential bacterial metabolite produced in the colon, since it is a preferred energy source for colon epithelial cells, contributing to the maintenance of the gut barrier function, as well as demonstrating immunomodulatory and anti-inflammatory capabilities [74,75]. Our results showed that the administration of DFC combined with centenarian-sourced *Lactobacillus casei* LTL1361 significantly increased butyrate and acetate concentrations in the colon contents of aged mice. Evidence indicates that butyrate and other SCFAs exert their effects through binding GPCRs that are involved in the resolution of inflammation in the gut, and GPR41 and GPR43 are the best-studied SCFA receptors [76,77]. This study found that the mRNA expressions of GPR41 and GPR43 were significantly upregulated in all treatment groups in the small intestine, whereas no significant difference was observed in colon. However, another study showed that the expression of GPR43 in the colon of aged mice was significantly decreased (*p* < 0.05) in a high-fiber diet (5% inulin) compared with a low-fiber diet (1% cellulose) for 4 weeks, but the authors did not investigate gene expression in the small intestine [76]. Furthermore, it is interesting to note that butyrate and acetate concentrations were highly positively correlated with serum T-AOC and IL-10, but negatively correlated with proinflammatory marker TNF-α. It is speculated that DFC combined with centenarian-sourced *Lactobacillus casei* LTL136 might ameliorate oxidative stress and systemic inflammation by elevating the production of SCFAs and upregulating the expression of SCFAs receptors.

## 5. Conclusions

As stated above, our study provided evidence that administration of the dietary fiber complex or centenarian-sourced *Lactobacillus casei* LTL1361 and their combination showed antiaging potential, associated with improved learning and memory ability, a protective effect of pyramidal nerve cells in the hippocampus, decreased oxidative stress and inflammation in serum and tissues, a protective effect of gut barrier function, and increased SCFA concentration and gene expression of SCFA receptors in the small intestine. Furthermore, the dietary fiber complex or *Lactobacillus casei* LTL1361 and their combination have the potential to become a promising functional food (probiotics or synbiotics) for the elderly. Further studies are required to clarify the detailed mechanism of the gut–brain axis in the metabolite-mediated antiaging effects.

## Figures and Tables

**Table 1:** Groups of naturally aging mice according to treatment method.

Group	Food	Oral Gavage
Control	Basal diet	0.9% saline
DFC	Basal diet added 10% DFC	0.9% saline
LTL1361	Basal diet	2 × 10^8^ cfu LTL1361/mice per day
DFC + LTL1361	Basal diet added 10% DFC	2 × 10^8^ cfu LTL1361/mice per day

**Table 2:** Gene-specific primers used for real-time PCR.

Bacteria	Genbank Access No.	Forward (F) and Reversed (R) Primer Sequence (5′–3′)	Product Length (bp)
Caspase-3	NM_009810.3	F: GTCATCTCGCTCTGGTACGG R: CACACACACAAAGCTGCTCC	169
Bcl-2	NM_009741.5	F: TACGAGTGGGATGCTGGAGA R: CGGTAGCGACGAGAGAAGTC	236
GPR41	NM_001033316.2	F: CGGCTCACTGTAGTGTGGTT R: AGTCGTACAGGCAGGAGGAT	127
GPR43	NM_001168509.1	F: TCCTTGATCCTCACGGCCTA R: TTGGATGCTGCTTCCACGAT	194
ZO-1	D14340.1	F: TGTGGATTTACCCGTCAGCC R: AGGACGGCCTCTTCCCTTAT	267
Claudin-1	NM_016674.4	F: CTCCTGTCCCCGGAAAACAA R: CAGAGGGAAGCAGCAGTTCA	311
β-actin	NM_007393.5	F: TACTGCTCTGGCTCCTAGCA R: CGGACTCATCGTACTCCTGC	146

**Table 3:** Escape latency of hidden-platform acquisition training test for five consecutive days.

Group	Escape Latency (s)				
	Day 1	Day 2	Day 3	Day 4	Day 5
Control	47.4 ± 7.3 ^a^	44.7 ± 6.4 ^a^	40.5 ± 5.6 ^b^	38.9 ± 8.0 ^b^	38.1 ± 4.7 ^b^
DFC	46.1 ± 7.6 ^a^	41.4 ± 7.5 ^a^	35.2 ± 6.6 ^ab^	31.0 ± 5.8 ^ab^	29.4 ± 6.7 ^a^*
LTL1361	43.2 ± 3.4 ^a^	38.5 ± 6.4 ^a^	35.3 ± 5.3 ^ab^	31.7 ± 5.1 ^ab^	27.2 ± 7.0 ^a^*
DFC + LTL1361	42.0 ± 7.6 ^a^	36.1 ± 10.7 ^a^	28.0 ± 5.6 ^a^	25.8 ± 7.0 ^a^	22.7 ± 6.9 ^a^**

All values are presented as the mean ± standard deviation (*n* = 6); * significant difference from the first day (*p* < 0.05); ** significant difference from the first day (*p* < 0.01); different superscript letters denote a significant difference in the same column (*p* < 0.05), according to one-way ANOVA.

## Data Availability

The data in this study are available on request from the corresponding author.
